# Meaning in Life and Loneliness as Mediators between COVID-19 Anxiety and Life Satisfaction in the Post-Pandemic among the General Population in Turkey: A Serial Mediation Model

**DOI:** 10.3390/ejihpe13100156

**Published:** 2023-10-09

**Authors:** Zafer Güney Çağış, Gülçin Güler Öztekin, Izaddin Ahmad Aziz, Francesco Chirico, Amelia Rizzo, Murat Yıldırım

**Affiliations:** 1 Department of Psychology, Faculty of Science and Letters, Mersin University, 33110 Mersin, Turkey; zaferguneycagis@gmail.com; 2 Department of Psychology, Faculty of Science and Letters, Agri Ibrahim Cecen University, Fırat Mahallesi Yeni Üniversite Caddesi No: 2 AE/1, 04100 Merkez/Ağrı, Turkey; ggoztekin@agri.edu.tr; 3 Special Education Department, College of Education, Salahaddin University-Erbil, Erbil 44002, Iraq; izaddin.aziz@su.edu.krd; 4 English Department, College of Education, Bayan University, Erbil 44002, Iraq; 5 Post-Graduate School of Occupational Health, Università Cattolica del Sacro Cuore, 00168 Rome, Italy; francesco.chirico@unicatt.it; 6 Health Service Department, Italian State Police, Ministry of the Interior, 00184 Milan, Italy; 7 Department of Clinical and Experimental Medicine, University of Messina, 98122 Messina, Italy; 8 Graduate Studies and Research, Lebanese American University, Beirut 13-5053, Lebanon

**Keywords:** COVID-19 pandemic, COVID-19 anxiety, meaning in life, loneliness, life satisfaction

## Abstract

The COVID-19 pandemic has impacted global society, leading to negative well-being and mental health outcomes. However, little is known about how COVID-19-related anxiety affects life satisfaction through psychological factors. This study examined the mediating roles of meaning in life and loneliness in the relationship between COVID-19 anxiety and life satisfaction in 333 Turkish general population (59.2% females; Mage = 33.9 ± 7.8). Participants completed measures of COVID-19 anxiety, life satisfaction, meaning in life, and loneliness. The results showed that COVID-19 anxiety predicted meaning in life, loneliness, and life satisfaction. Meaning in life predicted loneliness and life satisfaction, while loneliness predicted life satisfaction. Serial mediation analysis revealed that COVID-19 anxiety predicts life satisfaction through meaning in life and loneliness, even after controlling for age and gender. These findings contribute to our understanding of the underlying mechanisms between COVID-19 anxiety and life satisfaction, with implications for future research and practice.

## 1. Introduction

The rapidly spreading contagious COVID-19 disease caused by severe acute respiratory syndrome coronavirus 2 (SARS-CoV-2) adversely affects well-being and mental health outcomes [1,2]. Studies conducted in Turkey found that 19.0% of the participants exhibited high anxiety levels [3]. A recent systematic review also highlighted that approximately one-fourth of the participants reported experiencing mild to moderate anxiety during the COVID-19 pandemic. Furthermore, it was revealed that one in ten individuals dealing with anxiety may have a severe or even extreme anxiety disorder [4]. Recognizing the significance of providing support to individuals facing challenging circumstances, it is essential to note that higher satisfaction levels have been linked to lower anxiety levels [5]. Research showed that stay-at-home orders as a measure of the pandemic were associated with financial anxiety, greater health anxiety, and loneliness [6].

Furthermore, higher coronavirus anxiety was associated with more rumination and loneliness and lower subjective vitality [7]. Previous literature showed that the COVID-19 pandemic seriously threatened mental health, such as depression, anxiety, and fear [8,9]. In addition, recent studies have shown that the COVID-19 pandemic may have lasting effects on people’s well-being and mental health, and the risk of psychological health problems may increase over time [9,10,11]. However, psychological resources and strengths (e.g., meaning in life, hope, resilience, and optimism) were found to significantly contribute to individuals’ positive mental health and well-being in the face of adversity [1,12,13,14]. 

Life satisfaction, which is one of the pillars of subjective well-being, refers to the cognitive and judgmental process of one’s life in general, and this process is based on comparing how satisfied people are with their current situation according to individually selected criteria that are not imposed from outside [15]. The expanding literature has provided evidence showing a close link between life satisfaction and social support [16], self-compassion [17], mindfulness, self-esteem, and resilience [18], and negative relationships with mental health problems such as depression and anxiety [19]. In the context of the COVID-19 pandemic, life satisfaction was significantly negatively related to COVID-19-related fear and anxiety through psychological distress [20]. Similarly, a longitudinal study reported that COVID-19 was associated with deterioration in mental health by increasing symptoms of psychological distress, depression, and anxiety, as well as reducing life satisfaction [21]. 

The disturbing feeling that comes with the perception that one’s social needs are not met by the quantity or quality of social relationships is loneliness [22]. Loneliness leads people to negative consequences such as social isolation, coronavirus-related anxiety [23], poor sleep quality [24], psychological maltreatment [25], low life satisfaction, low quality of life [26,27], and poor well-being [28]. In the relevant literature, researchers have also revealed the mediating effect of loneliness on well-being and mental health outcomes. For example, loneliness exacerbates the association between coronavirus anxiety and rumination [29], COVID-19 peritraumatic distress, anxiety, and depression symptoms [30], perceived social support, depression, and anxiety [31], and rejection sensitivity and social anxiety [32]. On the other hand, a crucial predictor of loneliness is reduced meaning in life [33]. In other words, meaning in life allows people to regulate loneliness under challenging situations [34]. This emphasizes the importance of meaning in life, reducing loneliness, and supporting coping with adverse situations that may lead to serious mental health problems.

Frankl, one of the forerunners of Existential Theory, argues that people are characterized by an innate drive to find meaning in their lives. Life bestows a sense of purpose and significance to each individual; however, this sense of meaning can significantly vary from person to person and evolve [35]. Frankl’s perspective in logotherapy emphasizes not a universal, overarching meaning of life but rather the unique, moment-specific significance of an individual’s existence, often described as ‘healing through meaning’ [36]. According to Frankl [35], individuals can discover meaning in their lives through three avenues: creative values, experiential values, and attitudinal values. Attitudinal values entail the capacity to unearth meaning by adapting one’s attitude to inevitable adversity. People with a sense of meaning and purpose tend to persevere amidst challenges, demonstrating their resilience and survival [37].

Furthermore, it is important to note that a sense of meaning in life has positive implications for mental health, physical well-being, and overall wellness [37]. Therefore, it can be assumed that bolstering the perception of life’s significance among individuals experiencing COVID-19-induced anxiety could potentially mitigate feelings of loneliness and heighten satisfaction with life. This premise gives support for the foundation of our proposed mediation model in this study.

More importantly, Frankl notes in his autobiography that he increasingly realizes that life is immensely meaningful and that there must still be meaning even in suffering and failure. With this expression, Frankl emphasizes that stressful situations can be overcome by accepting life and developing a sense of meaning. In addition, meaning in life contributes positively to maintaining or improving mental health. Studies have provided evidence that meaning in life is positively associated with life satisfaction, better mental health, physical health, and well-being [38], and reduces stress, anxiety, and depression that emerge during adverse life circumstances [39]. Accordingly, meaning in life serves as a protective factor in improving well-being and mental health, as it mitigates adversity [13]. Kim et al. [40] found that the effect of depression was mediated by meaning in life, which provides a better quality of life. Some studies supported the mediating effect of meaning in life in the relationships between perceived stress and avoidant coping [41] and stressors and life satisfaction [42,43] by serving as a buffering effect against the negative consequences of stressful situations. The findings show that meaning in life provides a basis for assessing one’s chances of coping with life’s challenges. In light of previous studies, we can conclude that despite stress factors such as anxiety, depression, and stress that emerge over time and in different situations, people can function positively by returning to their essence of finding meaning in life, leading to well-being and life satisfaction.

The expanding literature mentioned above has provided evidence of an inverse relationship between anxiety and well-being due to unexpected situations such as the pandemic. It is essential to identify protective factors for better mental health. Thus, in this study with the Turkish general population, we suggest that high meaning in life and low loneliness may mitigate the negative impact of challenging situations on individuals’ well-being.

### Present Study

**Figure 1:**
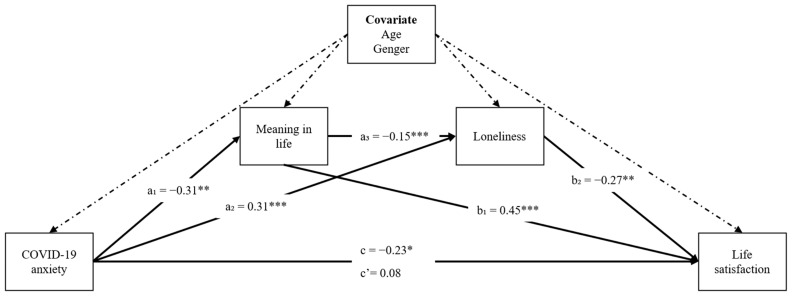
Serial mediation model with unstandardized coefficients. a_1_, direct effect of COVID-19 anxiety on meaning in life; a_2_, direct effect of COVID-19 anxiety on loneliness; a_3_, direct effect of meaning in life on loneliness; b_1_, direct effect of meaning in life on life satisfaction; b_2_, direct effect of loneliness on life satisfaction; c, total effect of COVID-19 anxiety on life satisfaction; c’, direct effect of COVID-19 anxiety on life satisfaction. * *p* < 0.05, ** *p* < 0.01, *** *p* < 0.001.

Although the roles of loneliness and meaning in life have been independently studied for COVID-19 anxiety and life satisfaction in some earlier studies, the simultaneous effect of these two variables concerning mental health and well-being outcomes has not been studied yet. Therefore, it is important to better understand the link between these variables to assist mental health professionals during the post COVID-19 pandemic. Based on the theoretical framework and empirical evidence documented above, this study aimed to examine meaning in life and loneliness as serial mediators in the relationship between COVID-19 anxiety and life satisfaction. To facilitate this aim, the following hypotheses were generated. The proposed serial mediation model is depicted in Figure 1.

**H1:**  *COVID-19 anxiety would be negatively associated with life satisfaction.*

**H2:**  *Loneliness would mediate the relationship between COVID-19 anxiety and life satisfaction.*

**H3:**  *Meaning in life would mediate the relationship between COVID-19 anxiety and life satisfaction.*

**H4:**  *Meaning in life and loneliness serially mediate the effects of COVID-19 anxiety on life satisfaction.*

## 2. Materials and Methods

### 2.1. Participants

Determining the adequacy of the sample size is important in research. It is commonly known that to detect an indirect effect among the variables of interest, a sample size ranging from 115 to 285 participants is typically required for 0.80 power to detect an effect [44]. In our study, a total of 333 Turkish young adults were recruited, which falls within this recommended range. This indicates that the sample size chosen for our study ensures sufficient statistical power to detect the desired effect. Table 1 presents the characteristics of the sample. Participants ranged in age between 18–63 years (M = 33.92, SD = 7.81), including 197 (59.2%) females. In total, 179 (53.8%) of the participants were married, 141 (42.3%) were single, and 13 (3.9%) were divorced/widowed. The majority of the participants were undergraduates (59.2%), followed by postgraduates (26.7%), and high school graduates (14.1). By percentage, 6.9% of the participants had low income, 81.4% had middle income, and 11.7% had high income.

### 2.2. Measures

The COVID-19 Anxiety Scale (CAS) was developed to assess physiological reactions of anxiety related to COVID-19 disease [8]. The CAS consists of 5 items (e.g., “I had trouble falling or staying asleep because I was thinking about the coronavirus”) rated on a 5-point Likert scale ranging from 0 = not at all to 4 = nearly every day over the last 2 weeks. A higher score on the CAS represents greater symptoms of coronavirus anxiety. The scale has excellent internal consistency reliability and a one-dimensional structure. Biçer et al. [45] adapted the CAS into Turkish. The internal reliability coefficient for the current study was 0.87.

The Meaningful Living Measure [46] is a self-report scale including 6 items (e.g., “I have meaningful social and close relationships”). All items are scored using a 7-point Likert scale ranging from 1 = strongly disagree to 7 = strongly agree. A higher score on the MLM suggests a greater level of meaning in life. It has strong internal consistency reliability. Cronbach’s alpha was 0.86 for the current study.

The UCLA8 Loneliness Scale [47] was used to assess participants’ feelings of loneliness. The scale is an 8-item self-reported scale answered on a 4-point Likert-type scale ranging from 1 (never) to 4 (often). An example statement is, “I am unhappy being so withdrawn”. The validity and reliability scale can be applied to Turkish culture [48]. The internal reliability coefficient for the current study was 0.78.

The Satisfaction with Life Scale [15] is a common measurement tool to assess the general life satisfaction of individuals. The SWLS is a one-dimensional scale consisting of 5 items (e.g., “In most ways, my life is close to my ideal”). All items are rated on a 7-point scale ranging from 1 = strongly disagree to 7 = strongly agree. Higher scores on the scale reflect greater satisfaction with life. The scale was adapted into Turkish by Durak et al. [49]. The Cronbach’s alpha for the present study was 0.85.

### 2.3. Procedure

After obtaining the approval of the university’s ethics committee, data were collected through an online survey and a convenience sampling method. Data were collected from the general population in Turkey. Consent was obtained from all participants before being involved in this study. A secure Google form link was generated and shared with participants to ensure data security. Participants were invited to this study through various communication tools (e.g., WhatsApp and Facebook). Participants were assured of anonymity and confidentiality about the responses and personal information. This survey took approximately 10 min to complete.

### 2.4. Data Analysis

The normality was assessed using skewness and kurtosis values, whereas Levene’s test was run to examine the homoscedasticity of variance. Pearson’s correlation coefficient analysis was performed to examine the relationship between variables. The mediating roles of meaning in life and loneliness in the relationship between COVID-19 anxiety and life satisfaction were examined using a serial mediation model tested using the PROCESS macro for the SPSS (Model 6). By controlling for age and gender as covariates in the analysis, 5000 bootstrap samples were used to estimate a 95% confidence interval. Confidence intervals that do not include zero indicate that the indirect effect is statistically significant [50]. All analyses were performed using the SPSS 20 and PROCESS macro for SPSS version 4.0.

## 3. Results

### 3.1. Preliminary Analyses

Descriptive statistics, Cronbach’s alphas, and correlation coefficients of the variables are presented in Table 2. All variables of this study were normally distributed. The skewness values were between ±3, and the kurtosis values were between ±10 [51]. Correlation analysis revealed that COVID-19 anxiety had a negative relationship with meaning in life (r = −0.20, *p* < 0.01) and life satisfaction (r = −0.13, *p* < 0.05), while it had a positive relationship with loneliness (r = 0.38, *p* < 0.01). On the other hand, meaning in life negatively correlated with loneliness (r = −0.36, *p* < 0.01) and positively correlated with life satisfaction (r = 0.58, *p* < 0.01), while loneliness was negatively correlated with life satisfaction (r = –0.34, *p* < 0.01). In addition, age was correlated only with life satisfaction (r = 0.15, *p* < 0.01), while gender was correlated with COVID-19 anxiety (r = 0.15, *p* < 0.01), meaning in life (r = −0.19, *p* < 0.01), loneliness (r = 0.14, *p* < 0.01), and life satisfaction (r = −0.23, *p* < 0.01).

### 3.2. Serial Mediation Analysis

Bootstrap sampling analysis presented significant serial mediation. We assessed COVID-19 anxiety as the independent variable (X), meaning in life as the first mediator (M1), loneliness as the second mediator (M2), and life satisfaction as the dependent variable (Y). Figure 1 displays the results of the mediation model that explores the role of meaning in life and loneliness in the relationship between COVID-19 anxiety and life satisfaction while controlling for age and gender.

As seen in Table 3 and Figure 1, the results of the analysis revealed that COVID-19 anxiety significantly predicted meaning in life (B = −0.312, SE = 0.10, t = −3.186, *p* < 0.01) and loneliness (B = 0.309, SE = 0.05, t = 6.446, *p* < 0.001). Similarly, meaning in life significantly predicted loneliness (B = −0.150, SE = 0.03, t = −5.651, *p* < 0.001). In addition, both meaning in life (B = 0.449, SE = 0.04, t = 10.559, *p* < 0.001) and loneliness (B = −0.267, SE = 0.08, t = −3.173, *p* < 0.01) were significant predictors of life satisfaction. Moreover, COVID-19 anxiety directly predicted life satisfaction (total effect; B = −0.235; SE = 0.06; 95% CI [−0.343, −0.120]. More importantly, after including mediators of meaning in life and loneliness in the analysis, the coefficient was insignificant (direct effect; B = 0.082; SE = 0.08; 95% CI [−0.070, 0.236]).

The analyses revealed that the indirect effect of COVID-19 anxiety on life satisfaction via meaning in life (X → M1 → Y) was significant (B = 0.014; SE = 0.05; 95% CI [−0.24, −0.04]). Similarly, the indirect effect of COVID-19 anxiety on life satisfaction through loneliness (X → M2 → Y) was also found to be significant (B = −0.08; SE = 0.03; 95% CI [−0.14, −0.03]). As a result of the serial mediating effect, the indirect effect of COVID-19 anxiety on life satisfaction through both meaning in life and loneliness (X → M1 → M2 → Y) was significant (B = −0.01; SE = 0.01; 95% CI [−0.03, 0.01]) (see Table 4).

## 4. Discussion

The current study used a relatively large Turkish sample to examine the association between COVID-19 anxiety and life satisfaction. We also examined the mediation effects of meaning in life and loneliness underlying the association. Consistent with our hypotheses, participants with higher levels of COVID-19 anxiety would experience decreased life satisfaction, with loneliness acting as a mediator. Furthermore, the serial mediation model (COVID-19 anxiety → meaning in life → loneliness → life satisfaction) produced a significant result, suggesting that individuals who experience more COVID-19 anxiety would report less meaning in life and experience more loneliness, thus leading them to experience poor life satisfaction. The findings are discussed in detail below.

Firstly, the findings showed that COVID-19 anxiety was negatively related to life satisfaction, supporting the first hypothesis of this study. Participants with COVID-19 anxiety reported low life satisfaction. These results were consistent with the findings of many previous studies [21,52]. A recent study with similar results found that life satisfaction negatively correlated with depression, anxiety, and stress during the COVID-19 pandemic [53]. In a study with university students in Turkey, participants reported symptomatic symptoms of depression, anxiety, stress, and post-traumatic stress disorder, which correlated in turn with poor psychological and physical health-related quality of life [54]. Surprisingly, some studies found a positive relationship between anxiety and life satisfaction during the pandemic [55]. One study, which was contrary to our results, found that COVID-19 fear and anxiety were positively associated with life satisfaction and assumed that this association was because while experiencing strict pandemic rules, those with high degrees of COVID-19 fear and anxiety were able to recognize the real values of life, such as being satisfied with their lives [20]. However, it should be further addressed in future studies to better understand the relationship between the two variables in stressful situations such as the COVID-19 pandemic.

Secondly, the results showed that loneliness partially mediated the association between COVID-19 anxiety and life satisfaction, providing evidence for the second hypothesis of this study. We determined that COVID-19 anxiety was positively associated with loneliness, which in turn was negatively associated with life satisfaction. Similarly, previous studies showed that loneliness mediated the effect of mental health and quality of life. For example, loneliness mediated the relationship between the negative impact of COVID-19 and quality of life [56]. Likewise, studies showed that those who perceived themselves as at risk of contracting COVID-19 had higher loneliness scores [57], and those who felt lonely faced significant threats to their well-being [58]. In a longitudinal study, after 12 months of follow-up, anxiety symptoms continued at high levels, depressive symptoms and loneliness increased, and life satisfaction decreased [59]. These results have provided evidence that situations beyond people’s control may have led to psychological consequences such as loneliness and low well-being.

Thirdly, the current findings yielded that meaning in life mediated the association between COVID-19 anxiety and life satisfaction, supporting this study’s third hypothesis. The current study provided evidence indicating that participants with COVID-19 anxiety reported lower meaning in life and life satisfaction. Also, meaning in life, which mitigated the negative effects of COVID-19 anxiety on life satisfaction, was a significant predictor of life satisfaction. Previous literature supported that individuals with positive feelings and a high sense of meaning in life were more likely to cope with negative life events and have better psychological health [7,12,60]. A study with Turkish people indicated the longitudinal predictive effect of meaning in life on young adults’ resilience and mental well-being during difficult times [61]. Moreover, past research showed that high meaning in life as a mediator was positively associated with various indicators of well-being and mental health [29,62]. For example, a study revealed that people who experienced COVID-19 stress were more likely to develop meaning crisis, which was associated with higher mental distress [63]. In addition to the studies in the literature, the results of our study show once again that meaning in life acts as a buffer between pandemic stressors and subjective well-being.

Finally, this study showed that meaning in life and loneliness serially mediated the effects of COVID-19 anxiety on life satisfaction, verifying the fourth hypothesis of the present study. In other words, individuals with high COVID-19 anxiety tended to have a lessor sense of meaning in life and a higher sense of loneliness, reducing their life satisfaction in Turkey. Previous findings are consistent with the results of this study regarding the relationship between COVID-19 anxiety, meaning in life, loneliness, and life satisfaction. For example, meaning in life and depression sequentially mediated the effects of family function on life satisfaction [64]. Another study indicated that increased meaning in life and life satisfaction resulted in lower anxiety and COVID-19 stress [43]. These findings have proven that meaning in life and life satisfaction allow people to regulate psychological factors on mental health and protect people’s mental health. Therefore, psychological resources and strengths support coping with adverse life situations such as pandemics that have the potential to cause serious mental health consequences and reduce anxiety, stress, depression, and loneliness.

The current study results indicate that in Turkish society, as in other countries, the COVID-19 pandemic has negative consequences on the well-being of individuals even in the post-pandemic period [65]. More importantly, we determine that high meaning in life and low loneliness mitigate this association under challenging times. Thus, we suggest that intervention programs based on these variables may be used preventively and therapeutically.

### 4.1. Contributions

The findings of this study with a Turkish sample contribute to the expanding literature and offer important implications for professionals dealing with COVID-19 anxiety, meaning in life, loneliness, and life satisfaction. When coping with COVID-19 anxiety, meaning in life acts as a factor that increases the level of life satisfaction and mitigates the level of loneliness. In contrast, loneliness serves as a factor that reduces the level of life satisfaction. In difficult times, our results provide evidence that individuals with anxiety may benefit from meaning in life. This is because anxiety negatively affects well-being by increasing the risk of psychological disorders and mental health problems [66,67,68,69], such as stress, depression, fear, loneliness, and post-traumatic disorders. Therefore, meaning in life is critical for fostering individuals’ mental health and might serve as a buffer for adverse consequences. In this regard, mental health professionals and psychological counsellors can help individuals reduce anxiety in stressful situations by reinforcing meaning in life and increasing life satisfaction, facilitating experiencing less loneliness in such situations. In addition, to promote well-being, mental health providers could consider the role of meaning in life and develop meaning-based interventions to prevent psychological stressors. As the findings of our study indicate that unexpected events such as the COVID-19 pandemic have psychological outcomes, it is of urgent importance that mental health professionals engage in practices that increase the preparedness and resilience of the general population.

### 4.2. Limitations

Although the present study offers important results, it nevertheless has some limitations. One significant limitation of this study pertains to the sample characteristics. The age range of the participants varied from 18 to 63 years old, with a mean age of 33.92 (SD = 7.81). The fairly even distribution of participants within this age range indicates a potential underrepresentation of individuals from the broader population. As such, this limitation restricts the extent to which this study’s findings can be generalized. To address this limitation, future research could consider exploring the study variables within distinct target groups, such as adolescents, young adults, and older adults. Alternatively, researchers may consider a more balanced representation of age groups within the sample. Additionally, replication of this study in diverse countries and cultural contexts would be valuable for better investigating the associations between the variables reported in this study. In addition, this study’s data were collected through an online survey via various communication tools, which leads to another limitation as this study has only encompassed internet users. Furthermore, despite the adequacy of the sample size (*n* = 333) for the analysis, it would be beneficial to consider replicating the current study utilizing a larger sample randomly selected from the target population. This will increase the generalizability of the findings, allowing for a broader application and enhanced external validity of the research outcomes.

## 5. Conclusions

In the post-pandemic period, people may hope that deterioration in mental health will decrease thanks to vaccines and measures. However, with the increase in the transmission of new variants, we are probably far from returning to the pre-pandemic situation [66,67,68,69]. Indeed, the available data showed that COVID-19 anxiety was linked to reduced meaning in life, leading to higher loneliness and lower life satisfaction as an indicator of the post-pandemic situation [70,71,72]. This result might reflect that life satisfaction further mitigates when COVID-19 anxiety leads to decreased meaning in life and increased loneliness. The current study also indicates the role of meaning in life and loneliness in improving life satisfaction. It suggests that focusing on the changes in life satisfaction is essential in difficult situations [73]. Mental health professionals can improve a sense of meaning in life by decreasing anxiety and loneliness, finally improving life satisfaction.

## Figures and Tables

**Table 1:** Characteristics of the sample (*n* = 333).

	Group	*n*	%
Gender	Female	197	59.2
	Male	136	40.8
Marital status	Married	179	53.8
	Single	141	42.3
	Widowed/divorced	13	3.9
Education level	High school graduate	47	14.1
	Undergraduate	197	59.2
	Postgraduate	89	26.7
Perceived socioeconomic level	Low	23	6.9
	Middle	271	81.4
	High	39	11.7

**Table 2:** Descriptive statistics, Cronbach’s alphas, and correlation coefficients of the study variables.

Variable	M	SD	*α*	1	2	3	4	5	6
1. Age	33.92	0.49	-	-	0.11 *	−0.02	0.07	−0.07	0.15 **
2. Gender	-	-	-		-	0.15 **	−0.19 **	0.14 *	−0.23 **
3. COVID-19 anxiety	1.34	0.58	0.87			-	−0.20 **	0.38 **	−0.13 *
4. Meaning in life	5.54	0.76	0.86				-	−0.36 **	0.58 **
5. Loneliness	1.74	1.57	0.78					-	−0.34 **
6. Life satisfaction	3.13	1.50	0.85						-

*Note*: * *p* < 0.05, ** *p* < 0.01; *α* = Cronbach alpha; M = mean; SD = standard deviation.

**Table 3:** Unstandardized direct estimates.

Predictor	Outcome	Coeff	SE	*t*	*p*
COVID-19 anxiety	Meaning in life	−0.31	0.10	−3.186	<0.01
COVID-19 anxiety	Loneliness	0.31	0.05	6.446	<0.001
Meaning in life	Loneliness	−0.15	0.03	−5.651	<0.001
Meaning in life	Life satisfaction	0.45	0.04	10.559	<0.001
Loneliness	Life satisfaction	−0.27	0.08	−3.173	<0.01
COVID-19 anxiety	Life satisfaction	0.08	0.08	1.071	>0.05

*Note*. SE: standard error; Coeff: Unstandardized coefficient.

**Table 4:** Unstandardized indirect estimates.

					95%	
Model Pathway	Mediator	Outcome	Coeff	SE	LLCI	ULCI
COVID-19 anxiety →	Meaning in life →	Life satisfaction	−0.14	0.05	−0.24	−0.04
COVID-19 anxiety →	Loneliness →	Life satisfaction	−0.08	0.03	−0.14	−0.03
COVID-19 anxiety →	Meaning in life → Loneliness →	Life satisfaction	−0.13	0.01	−0.03	−0.01

*Note*. SE: standard error; Coeff: Unstandardized coefficient; CI: Confidence interval; LL: Lower limit; UL: Upper limit.

## Data Availability

The datasets generated during and/or analyzed during the current study are available from the corresponding author upon reasonable request.
